# Heterogeneity of cell membrane structure studied by single molecule tracking†

**DOI:** 10.1039/d1fd00035g

**Published:** 2021-10-14

**Authors:** Gregory I. Mashanov, Tatiana A. Nenasheva, Alla Mashanova, Remigijus Lape, Nigel J. M. Birdsall, Lucia Sivilotti, Justin E. Molloy

**Affiliations:** The Francis Crick Institute 1 Midland Road London NW1 1AT UK Gregory.mashanov@crick.ac.uk Justin.molloy@crick.ac.uk; Koltzov Institute of Developmental Biology 26 Vavilova Str. Moscow 119334 Russia; School of Life and Medical Sciences, University of Hertfordshire, College Lane Hatfield Hertfordshire AL10 9AB UK; Department of Neuroscience, Physiology and Pharmacology, Division of Biosciences, University College London Gower St. London UK

## Abstract

Heterogeneity in cell membrane structure, typified by microdomains with different biophysical and biochemical properties, is thought to impact on a variety of cell functions. Integral membrane proteins act as nanometre-sized probes of the lipid environment and their thermally-driven movements can be used to report local variations in membrane properties. In the current study, we have used total internal reflection fluorescence microscopy (TIRFM) combined with super-resolution tracking of multiple individual molecules, in order to create high-resolution maps of local membrane viscosity. We used a quadrat sampling method and show how statistical tests for membrane heterogeneity can be conducted by analysing the paths of many molecules that pass through the same unit area of membrane. We describe experiments performed on cultured primary cells, stable cell lines and *ex vivo* tissue slices using a variety of membrane proteins, under different imaging conditions. In some cell types, we find no evidence for heterogeneity in mobility across the plasma membrane, but in others we find statistically significant differences with some regions of membrane showing significantly higher viscosity than others.

## Abstract 2

We use total internal reflection fluorescence microscopy combined with super-resolution tracking of multiple individual molecules, in order to create high-resolution maps of local membrane viscosity.

## Graphical abstract

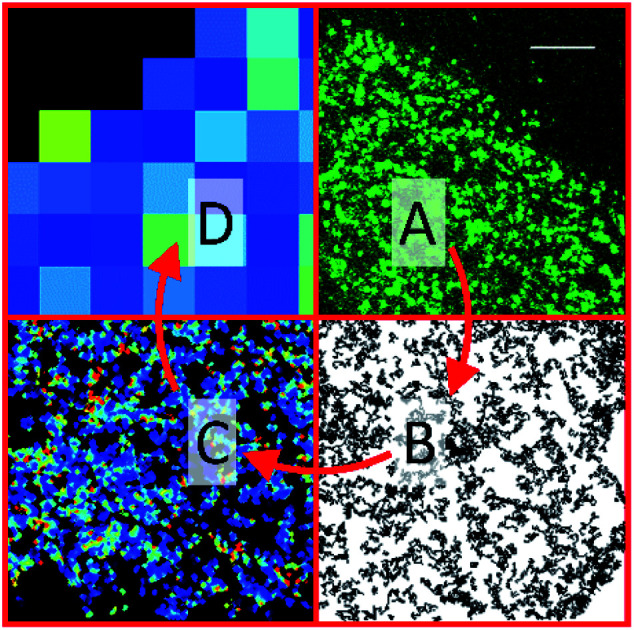

## Introduction

It is now thought that structural heterogeneity of the plasma membrane plays a critical role in a variety of cell functions.^1–2^ This contrasts with our classical view of the plasma membrane as a fluid mosaic bilayer of phospholipids and other amphipathic molecules within which proteins can diffuse in an unhindered manner.^3^ The path taken by a diffusing transmembrane protein should follow a random Brownian walk and a plot of its mean-squared displacement against time interval (MSD *vs.* d*T*) should be linear,^4^ with a gradient determined by *D*_lat_, the lateral diffusion coefficient. *D*_lat_ should be a linear function of membrane viscosity and thickness but should have only a weak dependence (*i.e.* log-function) on protein radius and viscosity of the bounding aqueous media (*i.e.* cytosol and extra cellular fluid).^5,6^ Recently a revised picture of the plasma membrane has emerged in which it is thought to have a more heterogenous structure compared to the classical fluid mosaic model, and there is biochemical and biophysical evidence for submicron, phase-separated, domains^7^ that are enriched with particular lipid species^8^ (“lipid rafts”) and regions of protein crowding or clustering. In addition, interaction of membrane proteins with the cortical cytoskeleton^9^ or extracellular matrix can lead to obstructions to free-diffusion^10^ (“picket-fences”). Together these features contribute to anomalous diffusive behaviour^11,12^ whereby MSD *vs.* d*T* plots are non-linear and show distinct downward curvature. The features that contribute to membrane heterogeneity are thought to be transient in nature and to extend over a length scale <200 nm, making them notoriously difficult to study in live cells. However, the advent of super-resolution, single molecule imaging methods^2,13^ allows “rafts”, “picket fences”, protein clustering and other heterogeneous properties to be studied in live cells and tissue slices in real-time.^14,15^ An advantage of single molecule studies is that they eliminate problems of bulk averaging that can mask short-lived, sub-resolution structural heterogeneities. Individual transmembrane proteins have been used as probes of membrane structure because they are of uniform size, can be labelled with high-specificity and known stoichiometry, they target to known membrane compartments and genetic manipulation allows structural alterations. However, tracking of single fluorophores in live cells is technically challenging because the spatial resolution and temporal precision of tracking is limited by photon emission rate and photobleaching of the fluorescent moiety. A useful figure of merit for a given fluorophore is the average number of photons emitted before photobleaching, giving rise to the idea of a “photon budget”.^16^ For a given experiment, best use of the photon budget requires a compromise to be made between tracking precision and measurement bandwidth. To measure small-scale, rapid movements, high laser power is required so that single fluorophores are bright and can be tracked with high-precision at fast video frame rate, but the cost is they soon photobleach. At low laser power, fluorophores can be tracked for longer but tracking resolution is reduced because fewer photons are emitted per second. It is also important to consider fluorophore density across the sample field of view (see ESI of ref. 14†) because at high density, fluorophore tracking becomes difficult as paths overlap, whereas at low density fewer molecules pass through a given region of interest making it difficult to accumulate sufficient data for statistical analysis. In general, fluorescent fusion proteins (like eGFP) photobleach faster than synthetic organic fluorophores (like rhodamine or Cy-dyes) and offer a lower photon budget.

The aim of the current study has been to test whether live mammalian cell membranes exhibit heterogeneity in viscosity on the micron length-scale. We have explored whether heterogeneity in protein mobility is evident in immortalised cell lines, primary cell culture and live tissue samples using different protein probes. Total internal reflection fluorescence microscopy (TIRFM)^17^ was used to image membrane proteins labelled with single fluorophores and studied their diffusive behaviour to infer local membrane properties. We used a quadrat sampling method that enables statistical tests to be performed on data obtained from different regions of the cell. Trajectories were segmented and diffusion coefficients computed from ≥10 data points (∼0.5 second) sections of each trajectory. The image of the cell was then sub-divided into a checkerboard pattern of sample quadrats (∼1–4 μm^2^ area each), and local statistics were calculated for trajectory segments that resided within each quadrat so statistical tests could be performed across different regions of the cell membrane. For most of the specimens tested, protein mobility was homogenous across the cell surface but in some cells we found evidence for spatial heterogeneity. We conclude that lipid rafts (if they exist) are evenly distributed across the plasma membrane of most cells, but in some cell types, they might show heterogeneity in density forming a “raft of rafts”.

## Experimental

All chemicals were obtained from Sigma-Aldrich, UK, unless stated otherwise.

### Cultured cell transfection and tissue labelling

All of the cells and tissue materials were imaged using a custom-made imaging chamber described previously.^18^ Human umbilical vein endothelial cells (HUVECs) (PromoCell GmbH, Heidelberg, Germany) derived from a pooled, primary cell culture were transfected with M_2_-eGFP muscarinic receptor fusion protein (kind gift of Prof. J. W. Wells, University of Toronto, Canada) using nucleofection according to the manufacturer’s instructions (Amaxa GMBH). CHO-K1 cells were transfected with cDNAs encoding the mouse α1, β1, and δ nicotinic receptor subunits subcloned within vector pRBG4 (kind gift of S. M. Sine, Mayo Clinic, MN, USA). The mouse γ-subunit was tagged with eGFP inserted into the cytoplasmic loop between transmembrane helices M3 and M4 and subcloned in pRK5 plasmid (kind gift of V. Witzemann, Max-Plank-Institute, Heidelberg, Germany). Primary cardiomyocytes were prepared as described previously^14^ and allowed to settle on to the imaging coverslip before labelling with Cy3B-telenzepine (10 nM for 1 h at 23 °C). The HL1 cardiomyocyte cell-line was cultured for 24 h before labelling with Cy3B-telenzepine (1 nM for 3.5 h at 23 °C). Zebrafish heart was removed and washed in room temperature PBS (pH 7.2) (Thermo Fisher Scientific, UK) solution supplemented with 10 U ml^−1^ heparin and 100 U ml^−1^ penicillin–streptomycin for 5 min. After washing, the heart was moved into an ice-cold “relaxing solution” consisting of PBS supplemented with; 1 mM EDTA, 2.5 mM KOH, and 3 mM MgCl_2_ for 5 min. A cutting block, with a 2 mm diameter cavity, and a single cutting-slot was used to bisect the heart along the ventricle axis.^15^ Mouse embryonic heart slices were prepared using procedures described previously.^14^ The prepared tissue slices were placed in relaxing PBS solution containing 10 nM of Cy3B-telenzepine for 1 h at 4 °C. This procedure labelled >95% of M_2_ muscarinic receptors with the tight-binding fluorescent ligand. The tissue slice was fixed against the coverslip with a fine nylon mesh grid (∼0.5 × 0.5 mm^2^) stretched in a stainless-steel tambour. The chamber was filled with Hank’s Balanced-Salt solution supplemented with 20 mM HEPES (pH 7.4). All imaging was performed at 23 °C.

### TIRF imaging

A custom-built TIRF microscope was used, as described previously.^19^ Briefly, the beam from a 100 mW, 488 nm laser or 150 mW, 561 nm laser (Light HUB-6, Omicron, Germany) was expanded using a Galilean beam expander and focused at the back focal-plane of a high numerical aperture, oil-immersion, objective lens (PlanApo, 100×, NA 1.45, Olympus, Japan) using a small, aluminium-coated mirror (3 mm diameter, Comar Optics, UK) placed at the edge of the back-aperture of the objective lens. The average laser power at the specimen plane was adjusted to ∼0.5 μW μm^−2^ and the incident laser beam angle was adjusted to ∼63° to create the evanescent field at the glass–aqueous medium interface. A second small mirror was placed at the opposite edge of the objective lens back-aperture to remove the returning (internally-reflected) laser beam from the microscope and a narrow band-pass emission filter FF01-525/50 or FF01-593/40 (Semrock, Rochester, NY) was used to block the scattered 488/561 nm laser light and other unwanted light. An EMCCD camera (iXon897BV, Andor, UK) captured video sequences at a rate of 20–50 fps, and the data were stored on a computer hard drive for analysis.

### Video data analysis

Video image sequences were analysed using GMimPro software (http://www.mashanov.uk), which employs an automatic single particle tracking algorithm (described previously^20^) to detect and track individual fluorophores. The position of each fluorescent spot was localised with sub-pixel resolution using a Gaussian fitting method and systematic drift was corrected *via* cross-correlation. Individual particle trajectories were output as a table of *x*,*y* coordinates measured at each video frame (*i.e.* each time point) for every fluorophore detected in the sample. From the tabulated data, the mean-squared displacement (MSD) of each fluorophore was computed over all possible time intervals (d*T*) (*e.g.* 1,2,3…*n* frames). MSD *vs.* d*T* plots were then generated to examine the particle motion; a linear-relationship is expected for simple Brownian motion, but curvature indicates anomalous diffusion (discussed later). To extract statistically meaningful mobility data from the trajectory analysis, we analysed trajectories where single fluorescent spots could be tracked for at least 10 consecutive frames with a permitted spot displacement of ≤0.7 μm between frames.^20^

### Single molecule mobility maps

We created a map to depict the lateral diffusion coefficient of our labelled protein probes by taking sequential 10-frame time windows (∼400 ms) along each single molecule trajectory and estimating the local *D*_lat_ value from the gradient of the MSD *vs.* d*T* plot. For display purposes, tracks were dilated to be 5 pixels wide (0.5 μm) and coded to produce a pseudo-colour heat map of membrane viscosity. At positions where trajectories overlapped, the pixel intensity was averaged. This approach is similar, but not identical, to single particle velocimetry.

### Measuring the local heterogeneity of cell membrane

We divided the image of each cell into a checkerboard pattern of sample quadrats and selected segments of single molecule trajectories that resided within each quadrat region. The local trajectory segments (at least 5 trajectories, ≥10 data points each per segment) were used to build an average MSD *vs.* d*T* plot. The initial slope of the plot was used to estimate the local *D*_lat_ (*i.e.* slope/4, for 2-dimensional diffusion). Local *D*_lat_ estimates were binned and histogrammed to build a global mobility map across the cell surface. The distribution of *D*_lat_ values of individual trajectories is described by a Gamma distribution due to the limited number of data points in each trajectory, but, according to the central limit theorem, the distribution of mean *D*_lat_ values measured at each quadrat sample should be normally distributed (*i.e.* Gaussian). We tested for heterogeneity across the checkerboard pattern of quadrat samples against an expectation based on the global distribution. The normality of the distributions was checked using the Kolmogorov–Smirnov (KS) test. The pairwise comparisons were done using Student’s *t*-test when both distributions were normal, and Mann–Whitney *U* (MW) test when at least one distribution was not normal.^21^ Calculations were performed using open-source *R* software.^22^

## Results

### Comparison of membrane viscosity in HL1 cell-line *vs.* primary cardiomyocytes

**Fig. 1:**
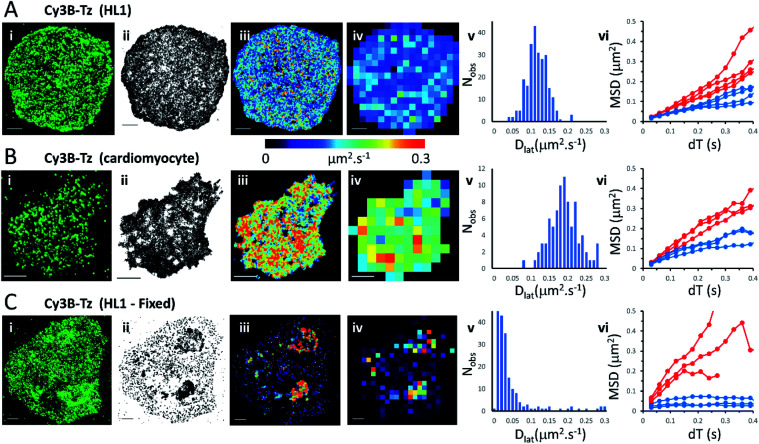
M_2_ muscarinic acetylcholine receptor diffusion on the plasma membrane of HL1 cells and primary cardiomyocytes. (A(i)) First video frame showing an HL1 cell-line with Cy3B-telenzepine labelled M_2_ receptors. (ii) A projection of all single molecule trajectory paths. (iii) Heat-map showing locally averaged *D*_lat_ values; mean *D*_lat_ = 0.12 μm^2^ s^−1^, *n* = 7526 trajectories. (iv) Heat map of the quadrat-sampled (2 × 2 μm^2^) *D*_lat_ values. (v) Distribution of *D*_lat_ measured in each quadrat region across the cell: 250 compartments, overall mean (of the local mean values) *D*_lat_ = 0.112 ± 0.028 μm^2^ s^−1^ (±SD). (vi) MSD *vs.* d*T* plots for typical fast (where *D*_lat_ > mean +1.5 × SD; red) and slow (where *D*_lat_ < mean −1.5 × SD; blue) quadrat regions. Video was recorded at 33 fps. (B) Panels as for A except: (i) primary cardiomyocyte. (iii) Mean *D*_lat_ = 0.203 μm^2^ s^−1^, *n* = 3133 trajectories. (v) Quadrats: 86 (2 × 2 μm^2^); mean *D*_lat_ = 0.188 ± 0.043 μm^2^ s^−1^. (vi) See panel A. (C) Panels as for A except: (i) HL1 cell chemically fixed with 1% paraformaldehyde. (ii) Trajectory paths show that most molecules have very restricted motion following fixation. Some regions show receptor diffusion (dark areas where molecular trajectories greatly overlap). (iii) Most M_2_ receptors are fixed, but some isolated regions show rapid receptor diffusion; average *D*_lat_ = 0.132 μm^2^ s^−1^, *n* = 6673 trajectories. (iv) Quadrats: 327 (2 × 2 μm^2^). (v) Distribution of *D*_lat_ values with overall mean *D*_lat_ = 0.029 ± 0.05 μm^2^ s^−1^. Fixed regions indicate the tracking noise floor (see main text and Fig. S1 C and D†). (vi) See panel A.

We first imaged HL1 cells, a cardiac muscle cell-line, that natively-express M_2_ muscarinic acetylcholine receptors (Fig. 1A and Movie 1†). Receptors were labelled using the tight-binding fluorescent ligand Cy3B-telenzepine^14^ under conditions that gave an optimal density of labelled receptors (∼0.8 receptors per μm^2^) for single molecule imaging and tracking purposes. Under the conditions used here, the Cy3B photobleaching rate was 0.19 s^−1^, (Fig. S1A†) which allowed us to track individual molecules for around 5 s. Many trajectories were prematurely truncated because fluorophores overlapped during a single frame. This caused one track to terminate and a new track to be generated when the fluorophores separated. A small number of, non-specifically, surface-bound Cy3B-telenzepine molecules were completely immobile throughout the video recording and these objects were removed from the dataset. The projected image of all single molecule trajectories shows the degree of coverage across the cell surface (Fig. 1A(ii) and S2†). The cell periphery has a higher track density because fresh (unbleached) fluorophores diffuse into this region from the apical cell surface which is beyond the evanescent field.

Analysis of individual trajectories (see methods) leads to estimates of *D*_lat_ for each molecule tracked and these values can be plotted as a pseudo-colour map (Fig. 1A(iii)) indicating M_2_ receptor mobility at different regions across the cell surface. To test for homogeneity, the mobility map is divided into a checker-board pattern of sample quadrats (Fig. 1A(iv)). Each quadrat is then colour-coded to represent the average *D*_lat_ value within that region. According to the central limit theorem the distribution of mean *D*_lat_ values should be Gaussian (Fig. 1A(v)), and in the following text “mean *D*_lat_” refers to the “mean of the quadrat means”. To investigate whether the diffusion of molecules in quadrats with extreme low or high *D*_lat_ values was anomalous we plotted MSD *vs.* d*T* diagrams from those, selected, quadrats (Fig. 1A(vi), S3A, B† and Table 1).

**Table 1:** Kolmogorov–Smirnov analysis of the local viscosity distributions^a^

Figure	Sample	Mean (μm^2^ s^−1^)	SD	*n*	K–S *D*-statistic	*p*-Value	Conclusion
Fig. 1	M_2_-Cy3B HL1	0.112	0.028	250	0.045	0.704	Normal
	M_2_-Cy3B primary myocyte	0.188	0.043	86	0.073	0.747	Normal
	**M**_**2**_ **-Cy3B HL1 fixed**	**0.029**	**0.050**	**327**	**0.283**	**<0.0001**	Not-normal***
	M_2_-Cy3B inter-cell HL1	0.129	0.015	29	0.121	0.793	Normal
Fig. 2	**M**_**2**_ **-eGFP-HUVEC**	**0.204**	**0.086**	**150**	**0.178**	**0.0002**	Not-normal***
Fig. 3	M_2_-Cy3B zebrafish cell 1 and cell 2	0.224	0.116	49	0.185	0.069	Normal
	Zebrafish cell 1	0.291	0.144	20	0.198	0.367	Normal
	Zebrafish cell 2	0.179	0.056	29	0.131	0.699	Normal
	**M**_**2**_ **-Cy3B mouse slice (all cells)**	**0.427**	**0.457**	**79**	**0.294**	**<0.0001**	Not-normal***
	Mouse slice cell 1	0.533	0.518	27	0.253	0.052	Normal
	**Mouse slice cell 2**	**0.534**	**0.590**	**18**	**0.370**	**0.010**	Not-normal**
	**Mouse slice cell 3**	**0.285**	**0.228**	**34**	**0.260**	**0.020**	Not-normal*
Fig. 4	**Nicotinic-eGFP-CHO**	**0.114**	**0.078**	**181**	**0.191**	**<0.0001**	Not-normal***
Fig. 5A	Model	0.188	0.034	227	0.046	0.721	Normal
Fig. 5B	**Model (both regions)**	**0.111**	**0.080**	**226**	**0.192**	**<0.0001**	Not-normal***
	Model (non-raft)	0.188	0.030	120	0.056	0.847	Normal
	**Model (raft region)**	**0.031**	**0.021**	**116**	**0.160**	**0.005**	Not-normal**

a Significance testing against normal distribution: *p-value < 0.05 (*e.g.* >95% confidence); **p-value < 0.01 ***p-value < 0.001.

We next imaged M_2_ receptors in primary cultured mouse cardiomyocytes that had been labelled the same way as the HL1 cells (Fig. 1B and Movie 2†). We found receptor diffusion was significantly faster (*D*_lat_ = 0.2 μm^2^ s^−1^) compared to the HL1 cell-line (*t*-test with unequal variances (*p* < 0.0001), *t*(111) = −15.4, *p* < 2.2 × 10^−16^), implying membrane viscosity was lower in the primary cultured cells. The molecular tracks were analysed as before and binned to give a checkerboard pattern of quadrat samples across the cell (Fig. 1B(i–vi) and Table 1).

In order to measure localisation and tracking errors (*i.e.* the “noise floor” of our measurements), we imaged HL1 cells that had been chemically fixed using 1% paraformaldehyde. Interestingly, after several minutes treatment with fixative, many cells still had isolated regions, or “islands”, of membrane that appeared sealed off from the fixative and where the Cy3B-telenzepine-labelled receptors continued to diffuse (Fig. 1C and Movie 3†). This fixation artefact gave the opportunity to generate diffusion maps in cells that showed extreme heterogeneity in receptor mobility due to incomplete fixation. Cell regions where chemical fixation was effective allowed individual molecules to be selected and tracked in order to give an estimate of the localisation and tracking error measured under our imaging conditions. The sum of all noise sources gave 33 nm root mean squared deviation (rms) (*i.e.* MSD = 1 × 10^−3^ μm^2^) (Fig. S1C and D).† However, the quadrat sampling method gave a three-fold higher estimate of the noise floor: rms ∼100 nm (MSD = 1 × 10^−2^ μm^2^) (Fig. 1C(v)). This difference arose because mobile molecules were often present within a given quadrat region. The quadrat mapping of *D*_lat_ values showed a heterogeneous distribution of values which is readily explained by the incomplete fixation, whereby some islands of plasma membrane seemed to be isolated and protected from the paraformaldehyde treatment. The mean *D*_lat_ values determined for each of the quadrat sample regions were histogrammed as before. The *D*_lat_ values showed an exponential distribution (Fig. 1C(iv)). Typical slow-moving quadrat regions (blue lines in Fig. 1C(vi)) exhibited MSD *vs.* d*T* plots that were highly anomalous, consistent with the molecules being either totally immobile or trapped in a confined area. The faster moving, outlier, *D*_lat_ values were from unfixed regions of membrane and MSD *vs.* d*T* plots (red lines in Fig. 1C(vi)) showed a variety of behaviours, with some quadrat regions showing a relatively linear slope of MSD *vs.* d*T* plots and others showing a distinct downward curvature consistent with anomalous diffusive behaviour (Table 1).

### Mapping membrane viscosity using eGFP-tagged M_2_ receptors

**Fig. 2:**
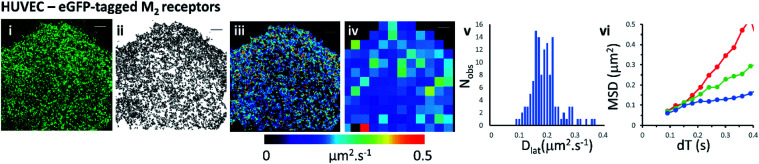
eGFP-tagged M_2_ muscarinic acetylcholine receptor diffusing at the plasma membrane of HUVECs. (i) Single image from the beginning of the record shows individual eGFP-tagged M_2_ receptors at the plasma membrane. (ii) Trajectory map; 5031 trajectories. (iii) Heat-map of *D*_lat_; mean *D*_lat_ = 0.197 μm^2^ s^−1^. (iv) *D*_lat_ quadrat map; 129; 4 × 4 μm^2^ quadrats (black indicates <5 trajectories, removed from later analysis). (v) Distribution of *D*_lat_ values; *D*_lat_ = 0.204 ± 0.086 μm^2^ s^−1^ (mean ± SD). (vi) MSD *vs.* d*T* plots for typical fast (where *D*_lat_ > mean +1.5 × SD; red), intermediate (where *D*_lat_ ≈ mean; green) and slow (where *D*_lat_ < mean −1.5 × SD; blue) quadrat regions. Video was recorded at 33 fps.

We next examined the mobility of a C-terminally-tagged M_2_-receptor eGFP-fusion protein where the fluorophore is positioned on the intracellular-side of the molecule in contrast to the extrinsic, synthetic labelling with Cy3B-telenzepine used in the previous experiments. HUVECs were transiently transfected with a recombinant M_2_-eGFP fusion construct in a mammalian cell expression vector and cells were then selected for imaging based on their receptor expression level (target level ∼0.8 receptors per μm^2^). The eGFP-tagged receptors moved rapidly and in a seemingly unhindered fashion across the plasma membrane (Fig. 2 and Movie 4†). The photobleaching rate of the eGFP fluorophore was 0.4 s^−1^ (Fig. S1B†) which is twice as fast as the Cy3B fluorophore. In addition to that, fluorophores could only be tracked for ∼0.5 s, because the eGFP signal-to-noise intensity ratio is lower than for Cy3B and this leads to premature track termination (*i.e.* tracking can fail before the fluorophore bleaches). Individual static molecules were selected and tracked in order to give an estimate of the localisation and tracking error measured under our imaging conditions. The sum of all noise sources gave 26 nm root mean squared deviation (rms) (*i.e.* MSD = 1 × 10^−3^ μm^2^) (Fig. S1C and D†), similar precision to Cy3B. The average rate of receptor diffusion (*D*_lat_ = 0.2 μm^2^ s^−1^) was similar to the primary cardiomyocytes (MW, *U* = 6265, *p* = 0.715) but the trajectories appear more compact (Fig. 2(ii)*vs.*Fig. 1A and B panel (ii)) simply because they are of shorter duration. The short tracks mean that there is less coverage (*i.e.* fewer unique tracks per unit area) when membrane viscosity maps are generated using a 4 × 4 μm^2^ quadrat size (Table 1).

### Comparison of Cy3B-telenzepine labelled M_2_ receptors in zebrafish tissue slice *vs.* mouse tissue slice

**Fig. 3:**
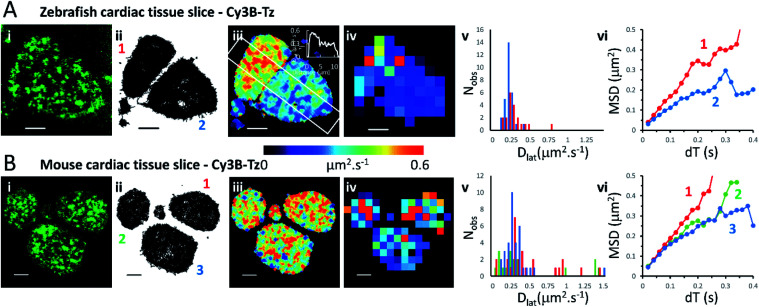
Cy3B-telenzepine labelled M_2_ muscarinic receptors in zebrafish and mouse cardiac tissue slices. (A(i)) First video frame showing individual Cy3B-telenzepine labelled M_2_ receptors on the plasma membrane of zebrafish cardiomyocytes in a tissue slice. (ii) Map showing the trajectory paths of individual molecules (see Movie 3).† (iii) Pseudo-colour heat-map showing locally averaged M_2_ receptor *D*_lat_ values. The overall mean *D*_lat_ = 0.33 μm^2^ s^−1^, 2518 trajectories. White rectangle shows profile plotted on the insert in the right-top corner. (iv) Heat map of the quadrat-sampled *D*_lat_ values; 49 quadrats (1 × 1 μm^2^) (black indicates <5 trajectories, removed from later analysis). (v) Distribution of *D*_lat_ measured in each quadrat region (red = “cell 1”, blue = “cell 2”): overall mean *D*_lat_ = 0.224 ± 0.116 μm^2^ s^−1^ (±SD): “cell 1” *D*_lat_ = 0.291 ± 0.144 μm^2^ s^−1^, “cell 2” *D*_lat_ = 0.179 ± 0.056 μm^2^ s^−1^. (vi) MSD *vs.* d*T* plots for typical “cell 1” (red), “cell 2” (blue) quadrat regions. (B) As for Panel A except: (i) mouse cardiomyocytes in a tissue slice. (iii) Mean *D*_lat_ = 0.428 μm^2^ s^−1^, 2336 trajectories. (iv) *D*_lat_ map: 83 quadrats (1 × 1 μm^2^). (v) Distribution of *D*_lat_ (“cell 1”(red); “cell 2” (green); “cell 3” (blue): mean *D*_lat_ = 0.427 ± 0.457 μm^2^ s^−1^). (vi) MSD *vs.* d*T* plots for typical quadrat regions (where *D*_lat_ ≈ global mean *D*_lat_); within “cell 1” (red); “cell 2” (green); “cell 3” (blue).

We investigated the M_2_ muscarinic receptor mobility in live tissue slices using two model cardiac systems (zebrafish and mouse), using Cy3B-telenzepine to fluorescently label the proteins. An initial observation was that the spread-area of the cells was noticeably smaller than for isolated primary cardiomyocytes and the HL1 cell-line; presumably because cells are held within the tissue and are less able to spread across the coverslip surface. Receptor density was similar in the tissue slices compared to the isolated cultured cells (∼1 receptor per μm^2^) (Fig. 3A, B and Movie 5† for comparison), but, the rate of diffusion was significantly faster in mouse tissue slices (mean *D*_lat_ = 0.43 μm^2^ s^−1^) compared to primary myocytes (MW, *U* = 1683, *p* = 2.3 × 10^−8^) and HL1 cell-line (MW, *U* = 2135, *p* < 2.2 × 10^−16^) and was similarly fast in the zebrafish tissue (mean *D*_lat_ = 0.33 μm^2^ s^−1^), but the median values were significantly different (MW, *U* = 1261, *p* = 0.001). Rapid receptor movement in both specimens produced a dense network of single molecule tracks so a small quadrat size (1 × 1 μm^2^) could be used and this gave a higher-resolution map of membrane viscosity (Fig. 3(iv)). A histogram of mean *D*_lat_ values derived from each quadrat sample was normally distributed (Fig. 3(v)). MSD *vs.* d*T* plots for three of the “slower” quadrat samples compared to 3 of the “faster” regions showed that MSD increased linearly with d*T* for both fast and slow samples and did not show substantial curvature (that would indicate anomalous diffusion) (Fig. 3(vi)).

Although the global average *D*_lat_ was significantly faster in tissue slices (*D*_lat_ = 0.33 μm^2^ s^−1^) than isolated cultured cells, the difference was smaller in the *D*_lat_ maps because faster trajectories were underrepresented and the mean *D*_lat_ value fell to 0.22 μm^2^ s^−1^ for the zebrafish tissue slices. Consistent with this, when quadrat size was increased to 2 × 2 μm^2^, the mean *D*_lat_ value increased to 0.27 μm^2^ s^−1^. Interestingly, we found significant differences in mobility between neighbouring cells (Fig. 3(iii)), implying that there are significant differences in membrane composition between cardiomyocytes within a given tissue sample. In the example shown here, one cell gave *D*_lat_ = 0.29 μm^2^ s^−1^ the other, *D*_lat_ = 0.18 μm^2^ s^−1^ (*t*-test with unequal variances, *t*(23) = 3.224, *p* = 0.004).

### Mapping membrane viscosity using nicotinic acetylcholine ion channels

**Fig. 4:**
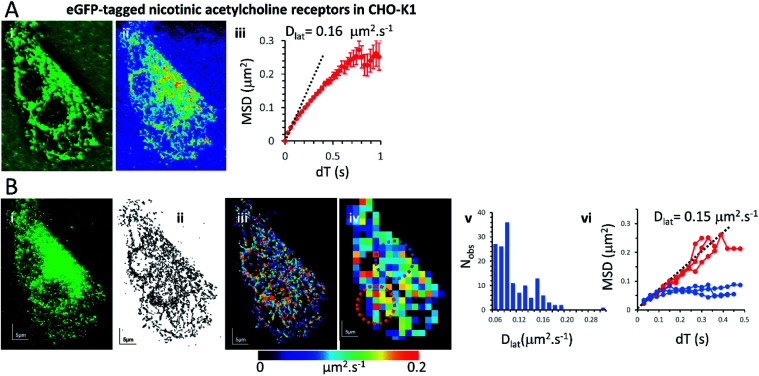
Nicotinic acetylcholine receptors were present at the plasma membrane and the ER of CHO-K1 cells: diffusion of nicotinic receptors was highly anomalous because the eGFP tagged γ-subunit only partially targeted the plasma membrane and the majority was found in the ER. (A(i)) Average projection of the video stack. (ii) Standard-deviation projection shows the reticulated network and also darker regions (presumably below the nucleus) where the ER was excluded and receptors could be seen moving at the plasma membrane. (iii) The overall MSD *vs.* d*T* plot showed evidence of anomalous diffusion with initial gradient (determined by least-squares’ linear regression) indicating *D*_lat_ = 0.16 μm^2^ s^−1^. (B(i)) First video frame showing single fluorophores. (ii) Projection of all the single fluorophore trajectories. (iii) *D*_lat_ map from trajectory segments. (iv) *D*_lat_ quadrat map (black indicates <5 trajectories, removed from later analysis). (v) Distribution of quadrat *D*_lat_ means (note that the histogram is not normally distributed). (vi) MSD *vs.* d*T* for fast (where *D*_lat_ > mean +1.5 × SD; red) and slow (where *D*_lat_ < mean −1.5 × SD; blue) regions. The fast-moving regions were located under the cell nucleus where receptors diffused freely at the plasma membrane (MSD *vs.* d*T* plots were linear with initial gradient (determined as in A) giving *D*_lat_ = 0.15 μm^2^ s^−1^). The slow-moving regions were from the network (ER) regions where receptors showed anomalous diffusion (note distinct downward curvature).

Our expectation was that the nicotinic acetylcholine receptor, which is a hetero-pentameric ion channel, should exhibit unrestricted diffusive motion at the plasma membrane. However, we found it did not localise strongly to the plasma membrane and most molecules were retained in the endoplasmic reticulum (ER) following transfection. This might arise because of its hetero-pentameric nature, comprising four different polypeptides; β1, δ, γ and two α1 subunits, that must assemble correctly following co-transfection of the α1, β1, and δ subunits, together with the eGFP-γ-subunit. It is likely that a fraction of eGFP-γ subunits failed to assemble correctly into the heteromeric complex and are retained in the ER. So, we see a mixture of fluorophores at the ER network and also at the plasma membrane. Receptors that had localised correctly to the plasma membrane were most evident beneath the cell nucleus where the ER was excluded. We tracked all fluorophores as before and generated mobility maps to investigate mobility across the cell (Fig. 4). Our data show, perhaps unsurprisingly, that the histogram of *D*_lat_ values derived from the quadrat maps is not normally distributed, and while MSD *vs.* d*T* plots produced from “fast-moving” quadrat regions (plasma membrane) are consistent with an unconstrained Brownian walk, “slow-moving” regions show anomalous diffusive behaviour (MSD *vs.* d*T* plots show a distinct downward curvature) because γ subunits are confined to the ER membrane network.

### Monte Carlo simulation of membrane protein random walks

**Fig. 5:**
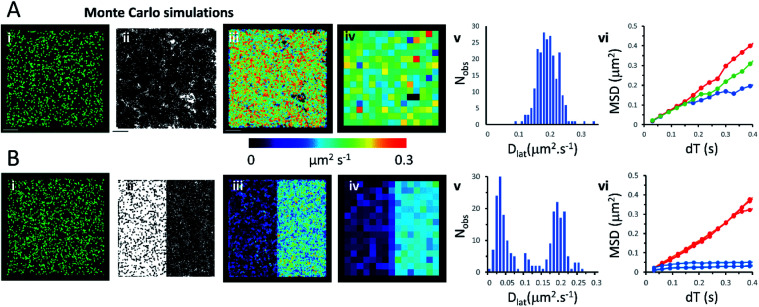
Monte Carlo simulation of single, fluorescently-tagged molecules moving at the plasma. (A(i)) Single image from the beginning of the simulated video recording shows individual fluorescent molecules (initial density 0.8 molecules per μm^2^). The simulated molecules are free to move in an unrestricted manner. (ii) Trajectory map consisting of 4434 trajectories. Fluorophore intensity, noise levels and photobleaching rate (0.2 s^−1^) were chosen to closely mimic our real data sets. (iii) *D*_lat_ map. (iv) Quadrat sampled *D*_lat_ map, 227 quadrats; 2 × 2 μm^2^, black squares contain <5 trajectories rejected from further analysis. (v) Histogram of quadrat *D*_lat_ values, is normally distributed and consistent with a single population. (vi) MSD *vs.* d*T* plots for typical fast (where *D*_lat_ > mean +1.5 × SD; red), intermediate (where *D*_lat_ ≈ mean; green) and slow (where *D*_lat_ < mean −1.5 × SD; blue) quadrat regions. (B) As for Panel A except: (i) the left-hand side of the simulation molecules are confined to lipid rafts; right-hand side molecules are free to move in an unrestricted manner, (ii) trajectory map, (iii) *D*_lat_ map, comprises two distinct regions. (iv) Quadrat sampled *D*_lat_ map. (v) Histogram of quadrat *D*_lat_ values, is now clearly bi-modal and not consistent with a single population. (vi) MSD *vs.* d*T* plots for typical fast (where *D*_lat_ > mean +1.5 × SD; red) and slow (where *D*_lat_ < mean −1.5 × SD; blue) quadrat regions. The “slow” (blue curves) regions show anomalous diffusive behaviour because molecules are trapped within modelled rafts of 300 × 300 nm^2^ area.

We used an object-based, Monte Carlo stochastic model^23^ to generate sequences of simulated TIRFM videos (Fig. 5 and Movie 6†). The random movement of single molecules were realistically reproduced with known intensity levels, stochastic shot-noise, background noise and photobleaching behaviour consistent with our experimental TIRFM imaging modality. The simulated video data sets were analysed with the same image processing software used for our real data sets. We first checked that our localisation and tracking software gave consistent results over a wide range of simulated *D*_lat_ values (see ESI Fig. S5†). The example simulation in Fig. 5A shows trajectories detected in a sequence of images simulating molecules moving at *D*_lat_ = 0.2 μm^2^ s^−1^. The *D*_lat_ map (Fig. 5A(iii)) appears similar to our cell imaging data (Fig. 1A, B, 2 and 3). The local *D*_lat_ map calculated for a 2 × 2 μm^2^ quadrat size showed a normal distribution of diffusion coefficients (Fig. 5A(v)). Note here, that any superficial appearance of heterogeneous behaviour is simply due to statistical variation across sample quadrats, and demonstrates the necessity for statistical tests of heterogeneity. In fact, there is no deviation from normality (Table 1).The MSD *vs.* d*T* plots (Fig. 5A(vi)) were linear for the “fast”, “intermediate” and “slow” quadrat samples (note: we define “fast” as *D*_lat_ > mean +1.5 × SD, “slow” *D*_lat_ < mean −1.5 × SD and “intermediate” as *D*_lat_ ≈ mean). We also simulated the presence of lipid raft regions 300 × 300 nm^2^, dispersed randomly across the membrane (Fig. 5B, on the left-hand side of the modelled membrane) in which molecules were confined (*D*_lat_ = 0.02 μm^2^ s^−1^) and these regions adjoined others where molecules diffused freely with *D*_lat_ = 0.2 μm^2^ s^−1^ (on the right-hand side of the membrane). Now, the distribution of quadrat mean *D*_lat_ values were no longer normally distributed (Fig. 5B(v)). “Slow” regions showed anomalous diffusion with downward curvature, because molecules were confined within the modelled “lipid rafts”, whereas “fast” regions had linear MSD *vs.* d*T* plots.

## Discussion

In the spirit of Faraday meetings our intention is to provoke discussion on the structure of biological membranes: the classical view of the plasma membrane is that it comprises a fluid-mosaic of lipids, proteins and other amphipathic molecules.^3^ This view has been challenged and it is now proposed that there is structural heterogeneity arising from lipid de-mixing and phase-separation which results in the formation of sub-micron sized microdomains or “lipid rafts”, enriched with saturated lipids, sphingolipids and cholesterol.^24^ Our view of the plasma membrane has therefore changed from a fully-mixed fluid-mosaic sheet to a cholesterol- and protein-stabilised, oil-in-oil, emulsion that resembles a two-dimensional “mayonnaise”. The small-size and short-lived, nature of lipid microdomains has led to controversy^25–27^ and as technologies have improved, microdomain size has fallen from 1 micrometre to as small as 10 nm diameter. Although phase separation has been demonstrated in model lipid systems^7,28^ there are few, direct observations of heterogeneity in live mammalian cells at physiological temperature.^29,30^ In a previous study, we found the mobility of M_2_ receptors in HL1, CHO-K1 and primary cardiomyocytes showed no evidence of a phase transition over a −5 °C to +45 °C temperature range.^14^ Stability of lipid microdomains may be impacted by in-plane, protein–protein and protein–lipid interactions and there may be additional interactions that extend out of the membrane plane onto the intracellular cytoskeleton and/or extracellular matrix. These interactions may act to stabilise raft structures and corral or otherwise interfere with the free diffusion of membrane proteins^10^ which may either partition into the raft or the surrounding isotropic lipid, or may “hop” between the two regions.^31^

Notwithstanding the controversy surrounding the existence of lipid rafts and membrane microdomains, their proposed presence is thought to affect both the distribution and mobility of transmembrane proteins, with important consequences in neurobiology, virology, immunology and membrane–peptide interactions. One manifestation of membrane heterogeneity is that diffusive motion of transmembrane proteins becomes anomalous and MSD *vs.* d*T* plots are non-linear and show distinct downward inflection, indicating that molecules diffuse rapidly over short time and length scales, but more slowly over longer time intervals and distances.^12,28,32^ A significant problem with use of MSD *vs.* d*T* plots is that raw data derived from single molecule imaging experiments rarely enables meaningful analysis to be made on a single molecular trajectory.^11,33^ In earlier work, we found that muscarinic acetylcholine receptors (a class of membrane-spanning, 7-helix, G-protein coupled receptor) undergoes unrestricted diffusion at the plasma membrane.^34^ So, we chose this protein as a prototype probe of membrane viscosity and structural heterogeneity. Here, we have investigated whether molecules diffuse at different speeds in different regions of the plasma membrane and whether in regions where molecules appear to move more slowly they also exhibit anomalous diffusive behaviour. We divided the cell membrane into 1 × 1 μm^2^ or 2 × 2 μm^2^ quadrats and analysed (≥5) independent molecular trajectories in each quadrat region so we could test for significant variation between cell membrane regions. We also tested if the mean *D*_lat_ values estimated at each quadrat were homogeneous and normally distributed, as expected by the central-limit theorem. We then specifically compared MSD *vs.* d*T* plots from “slow” and “fast” moving regions to see if the plots were linear or showed evidence of anomalous diffusive behaviour.

Measurement precision to some extent depends on imaging conditions, including the type of cell, choice of membrane protein and the fluorescent tag that has been employed. The density of single molecule trajectories (or “tracks”) and the spatial resolution of local *D*_lat_ maps, depends on the lateral diffusion (*D*_lat_) and track duration; which are limited mainly by the fluorophore photobleaching rate. To generate a local *D*_lat_ estimate we constructed MSD *vs.* d*T* plots from single particle tracks that extended for ≥10 consecutive video frames.^35^ Excitation laser power and imaging rate were optimised to allow molecules to be unambiguously tracked with high spatial and temporal resolution over a sufficient number of video frames. The density of unique single molecule trajectories that were accumulated over the entire imaging period (∼1 min) varied between samples even though the starting fluorophore density was similar (∼1 molecule per μm^2^). For most specimens a quadrat size of 2 × 2 μm^2^ was used, but some (with high track density) allowed the use of a smaller quadrat size (1 × 1 μm^2^). For randomly moving molecules (free diffusion), a histogram of *D*_lat_ values obtained by fitting all of the individual single molecule MSD *vs.* d*T* plots over the entire cell surface is best described by a gamma distribution.^11,36^ However, we show here that a histogram of mean *D*_lat_ values, obtained by averaging individual tracks within each quadrat sample area, obeys the central limit theorem and shows a normal distribution. Departure from normality implies heterogeneity in the data set at the level of the quadrat sample size; this can be assessed by Kolmogorov–Smirnov analysis (summarised in Table 1).

We found that the diffusive motion of M_2_ receptors at the plasma membrane of HL1 cells, primary cardiomyocytes and zebrafish cardiac tissue slices all gave pooled MSD *vs.* d*T* plots (*i.e.* taking all molecular trajectories) that were linear (Fig. S4†), with no obvious evidence of anomalous diffusion. When trajectories were segmented and subdivided into checkerboards of quadrat samples, the histograms of mean *D*_lat_ values were found to be normally distributed. Thus, there was also no evidence for heterogeneity in receptor mobility across the plasma membrane of these specimens. When we further analysed the data by examining MSD *vs.* d*T* plots on quadrat sample trajectories drawn from seemingly “fast” and “slow” moving regions we found the plots have no obvious downward curvature. Together these findings are consistent with variation in individual *D*_lat_ values, being the result of random sampling of a homogeneous population. So, in these cases, lipid rafts must not cause anomalous diffusive motion and/or must be monodisperse and evenly distributed across the plasma membrane (see Table 1 and Fig. 1–3).

We found three specimens that convincingly exhibited *D*_lat_ histograms that were not normally distributed; implying that the quadrat samples were not drawn from a simple homogeneous population. We were interested to compare our findings for nicotinic acetylcholine receptors with another recent study^37^ in which ion channel motion was tracked in frog embryonic muscle fibres using quantum dot labels and where MSD *vs.* d*T* plots were essentially linear once immobile objects were removed from the data sets and MSD *vs.* d*T* plots were essentially linear. However, an increasing deviation from unity gradient for log(MSD) *vs.* log(d*T*) plots at shorter times and smaller distances, indicated the receptors were diffusing in an anomalous manner that was best fit by an exponential distribution of diffusion coefficients. The authors point out that a distribution of diffusion coefficients is inconsistent both with simple lipid raft or cytoskeletal picket fence model. The same type of effect was seen in another high-resolution, quantum-dot tracking study.^38^

In the current study, we found downward curvature of the MSD *vs.* d*T* plots when we analysed data collected across the whole CHO-K1 cells transfected with the eGFP-tagged γ-subunit and all other component polypeptides of the nicotinic receptor. This indicates a highly anomalous diffusive behaviour of the ion channel. However, the explanation for our finding is rather simple because a standard-deviation projection of the video data revealed that many of the eGFP-tagged subunits were associated with the ER, and when regions that were rich in ER were examined separately (using our quadrat sampling method), diffusion was slow and highly anomalous. However, regions of the cell where the ER was excluded (*e.g.* beneath the nucleus) the ion channels moved rapidly and exhibited linear MSD *vs.* d*T* plots. The rate of diffusion in the fast-moving regions was 10× greater than values reported in the earlier study using frog muscle cells.^37^

We show that data averaging between the cells should be applied with caution, or avoided altogether, because variation in viscosity between the cells can be higher than variations within a single cell (see the viscosity map profile values on Fig. 3A(iii)). The *D*_lat_ quadrat distributions found between two neighbouring myocytes have very different K–S test scores when considered either separately or together (Fig. 3A(iv) and Table 1). Also, membrane heterogeneity within an individual endothelial cell expressing eGFP-tagged M_2_ receptors was evidenced by the “non-normal” distribution of local viscosity values (Fig. 2(v) and Table 1). Visual inspection of the quadrat map (Fig. 2(iv)) revealed that mobility in the lower-left region of the cell was significantly lower than for the rest of the cell.

## Conclusion

We have presented a method to analyse single fluorophore tracking data, which employs a quadrat sampling technique to partition data into localized maps of transmembrane protein mobility. We have shown that, in many cases, the plasma membrane has a uniform viscosity and homogeneous structure, and quadrat mean values are normally distributed as expected by the central limit theorem. However, and perhaps as expected, proteins that localised to two different membrane systems (nicotinic receptors found at the ER and plasma membrane or CHO-K1 cells) or following incomplete chemical fixation (M_2_ receptors in HL1 cell) showed distinct heterogeneity. In one specimen, M_2_ receptors in an endothelial cell, we found spatial variation in membrane viscosity across an individual cell, and while the bulk MSD *vs.* d*T* plot was perfectly linear (Fig. S3B†) the quadrat mean *D*_lat_ values were not normally distributed (Table 1). Closer inspection of the MSD *vs.* d*T* plots for “fast” and “slow” diffusing regions showed the difference in mobility was not due to anomalous diffusion (*i.e.* microscopic variation in structure) but more likely to a bulk variation in membrane viscosity (a “raft of rafts”) that impacts the macroscopic *D*_lat_ value but has minimal effect on microscopic, anomalous diffusive motion of individual M_2_ receptors.

## Conflicts of interest

There are no conflicts to declare.

## Supplementary Material

FD-232-D1FD00035G-s001

FD-232-D1FD00035G-s002

FD-232-D1FD00035G-s003

FD-232-D1FD00035G-s004

FD-232-D1FD00035G-s005

FD-232-D1FD00035G-s006

FD-232-D1FD00035G-s007
