# Supplementary material for: Heterogeneity of cell membrane structure studied by single molecule tracking
Source: Faraday Discuss. 2021 Oct 14;232:358–74. doi: 10.1039/d1fd00035g (PMC8704140; doi:10.1039/d1fd00035g)
Supplement: FD-232-D1FD00035G-s007 [file FD-232-D1FD00035G-s007.pdf]

Electronic Supplementary Material (ESI) for Faraday Discussions.
This journal is © The Royal Society of Chemistry 2021

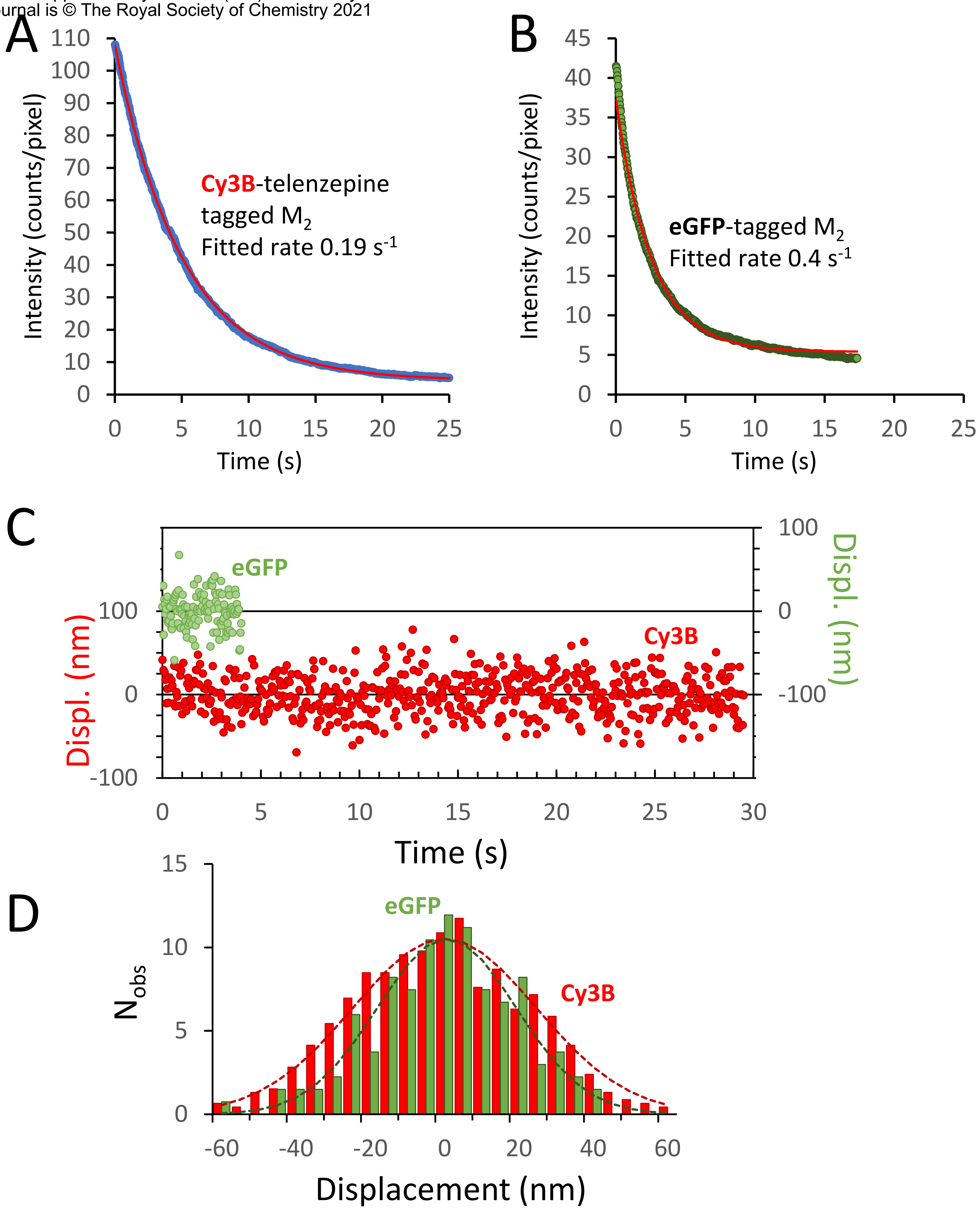

Fig S1. Photobleaching rate and tracking accuracy: A) M_2 receptors labelled with Cy3B-telenzepine in an HL1 cell **(from Fig. 1Ai main paper).** Blue dots raw data, red line fitted exponential 0.19 s^{-1}. **B)** M_2 receptors tagged with eGFP in a HUVEC cell **(from Fig. 2Ai, main paper)** Green dots raw data; red line fitted exponential 0.4 s^{-1}. **C)** Representative single fluorophore positional noise in a fixed HL1 cell labelled with telenzepine-cy3B (red, fluorophore bleaches after ~29s) and eGFP-tagged M2 receptors in a HUVEC cell (green, fluorophore bleaches after ~4s). Video was recorded at 30fps. Note that the positional variance is the sum of all noise sources and gives a "worst case" tracking accuracy (i.e. it includes positional noise due to mechanical vibration, etc). **D)** Histograms of fluorophore displacement values measured in **(C)** Gaussian fit with RMS deviations Cy3B = ±33nm: eGFP= ±26nm.

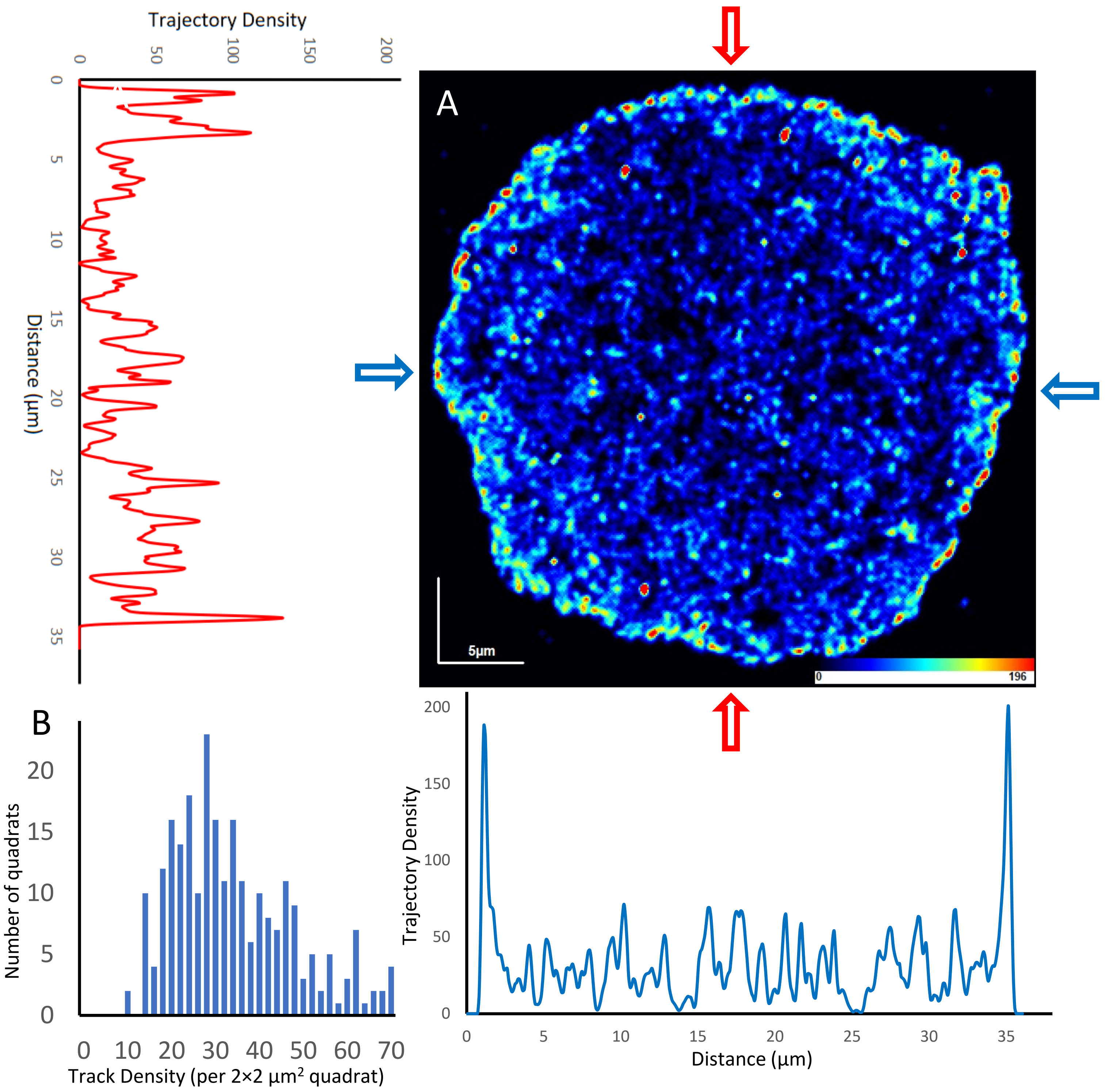

Fig. S2. Trajectory density analysis. **A)** Pseudo-colour trajectory density map for the cell shown in the main text **Fig. 1Ai** (Cy3B-telenzepine bound to M_2 receptors in an HL1 cell). Each x,y data point in the trajectory was dilated to a diameter of 0.5 µm and the pixel values were summed to represent the number of overlapping trajectories and displayed using a pseudo-colour look-up table (here in the range 0->196 overlapping tracks per pixel). Two profiles, 0.5 µm wide, were drawn though the centre of the cell and are shown at the edges of the image: blue – horizontal, red – vertical. **B)** Histogram to show the distribution of trajectory densities across the quadrat checkerboard (2×2 µm^2) (see the main text **Fig. 1Aiv**). The average trajectory density was 34.6 per quadrat (note zero values were excluded).

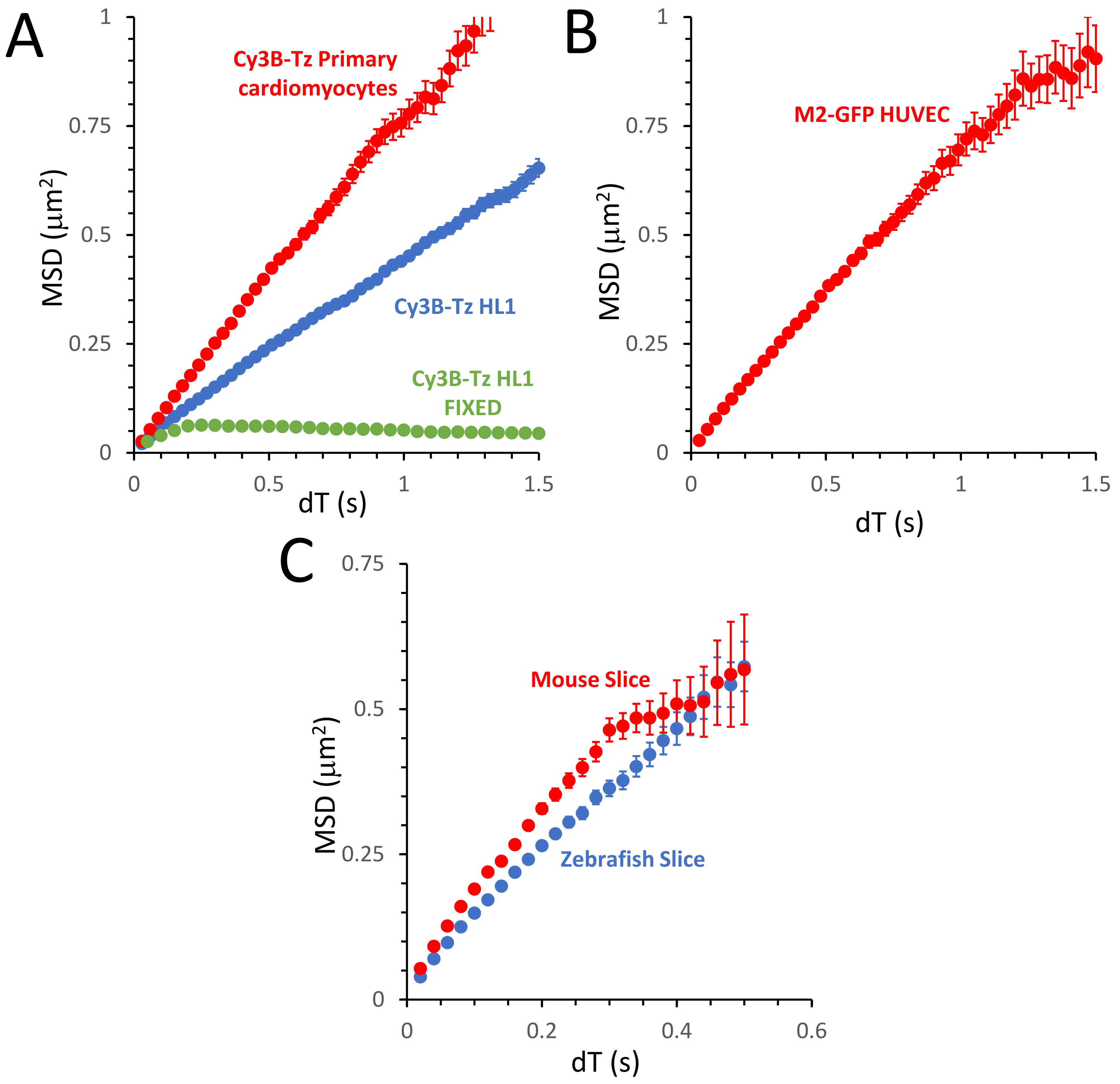

Fig S3: Mean Squared Displacement vs. time interval (dT) plotted for all molecular trajectories for entire video records: A) Data sets for cells shown in the main text **Fig. 1 B)** Data sets for cells shown in the main text **Fig. 2**. **C)** Data sets for cells shown in the main text **Fig. 3**. (different scale from **A & B**). None of the plots show evidence for anomalous diffusion and they do not deviate very significantly from a straight-line relationship; Except panel **(A)** "Green" data for the chemically-fixed HL1 cell.

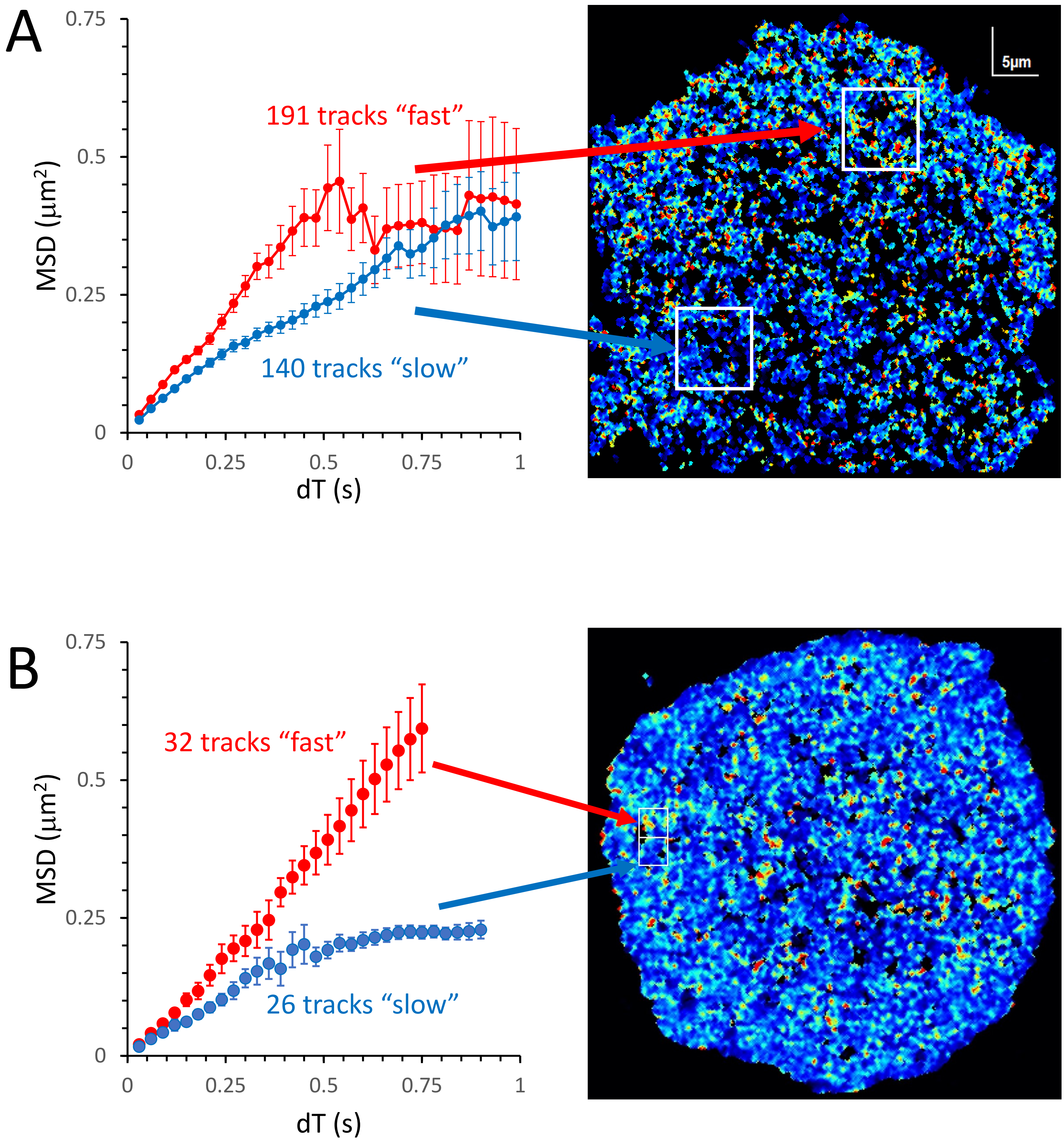

Fig. S4: Local variation in mean D_{lat} values are further analysed by inspecting the MSD vs dT plots for representative "fast" and "slow" quadrat regions: A) HUVEC cell. Left Panel: MSD-dT plot shows significant differences in M_2-GFP mobility in two separate 8×8 μm^2 regions of the cell marked by white rectangles on the **right panel.** The measured D_{lat} (fitted slope of MSD-dT plot, first 20 data points) was 0.208 $\mu m^2\ s^{-1}$ (191 trajectory averaged, red quadrat) and 0.111 $\mu m^2\ s^{-1}$ (140 trajectory averaged, blue quadrat). **Right Panel:** The enlarged heat map (**Fig. 2iii** main paper) showing locally averaged D_{lat} values. Two quadrats with substantially different viscosity values marked by the white rectangles. **B) HL1 cell. Left Panel:** MSD-dT plot shows significant differences in Cy3BTz labelled M_2 receptor mobility in two separate 2×2 μm^2 regions of the cell marked by white rectangles on the right panel. The measured D_{lat} (fitted slope of MSD-dT plot, first 20 data points) was 0.192 $\mu m^2\ s^{-1}$ (32 trajectory averaged, red) and 0.113 $\mu m^2\ s^{-1}$ (26 trajectory averaged, blue). Note the slow trajectories show anomalous diffusion (indicated by downward curvature). **Right Panel:** The enlarged heat map showing locally averaged D_{lat} values. The two respective quadrats with substantially different viscosity values are marked by white rectangles.

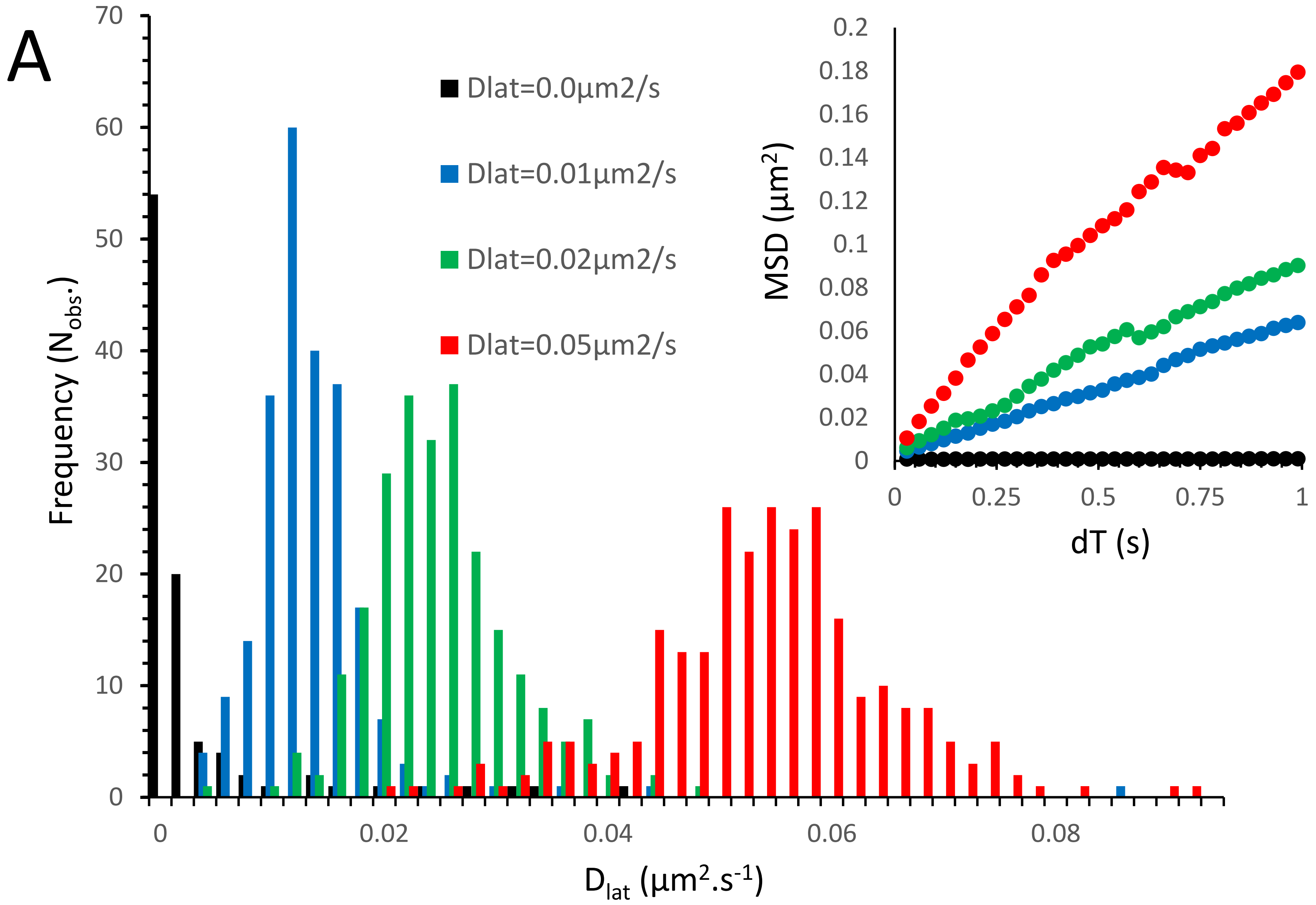

Fig. S5. Computer Simulations of single molecule diffusion with extremely low values of D_{lat}: Video data was simulated using an object-based*, Monte Carlo* random walk for fluorophores that have realistic intensity and signal-to-noise. The mock videos were then tracked and analysed in the same way as the real data sets from our TIRF imaging experiments. The D_{lat} quadrat mapping shows the distribution of mean D_{lat} values are normally distributed (i.e. obey the central limit theorem) except for the very slowest example which is dominated by tracking noise and where data under-sampling and histogram binning lead to artificial truncation near zero (black data).
